# Increased phosphorylation of histone H3 at serine 10 is involved in Epstein-Barr virus latent membrane protein-1-induced carcinogenesis of nasopharyngeal carcinoma

**DOI:** 10.1186/1471-2407-13-124

**Published:** 2013-03-18

**Authors:** Binbin Li, Guoliang Huang, Xiangning Zhang, Rong Li, Jian Wang, Ziming Dong, Zhiwei He

**Affiliations:** 1 Department of Pathophysiology, Basic Medical College of Zhengzhou University, No.100 of Science Road, Zhengzhou, 450001, China; 2 Key Laboratory for Medical Diagnostics of Guangdong Province, Sino-American Cancer Research Institute, Guangdong Medical College, No. 1 Xincheng Road, Guangdong, Dongguan, 523808, China; 3 Department of Pathophysiology, Guangdong Medical College, Guangdong, Dongguan, 523808, China

**Keywords:** Histone H3 phosphorylation, Nasopharyngeal carcinoma, Epstein-Barr virus latent membrane protein-1, MSK1

## Abstract

**Background:**

Increased histone H3 phosphorylation is an essential regulatory mechanism for neoplastic cell transformation. We aimed to explore the role of histone H3 phosphorylation at serine10 (p-H3Ser10) in Epstein-Barr virus (EBV) latent membrane protein-1 (LMP1)-induced carcinogenesis of nasopharyngeal carcinoma (NPC).

**Methods:**

The expression of p-H3Ser10 was detected by the immunohistochemical analysis in NPC, chronic nasopharyngitis and normal nasopharynx tissues, and its correlation with LMP1 was analyzed in NPC tissues and cell lines. Using the small interfering RNA (siRNA)-H3 and histone H3 mutant (S10A), the effect of histone H3 Ser10 motif on LMP1-induced CNE1 cell proliferation, transformation and activator protein-1 (AP-1) activation were evaluated by CCK-8, focus-forming and reporter gene assay respectively. Mitogen- and stress-activated kinase 1 (MSK1) kinase activity and phosphorylation were detected by *in vitro* kinase assay and western blot. Using MSK1 inhibitor H89 or siRNA-MSK1, the regulatory role of MSK1 on histone H3 phosphorylation and AP-1 activation were analyzed.

**Results:**

Immunohistochemical analysis revealed that the expression of p-H3Ser10 was significantly higher in the poorly differentiated NPC tissues than that in chronic nasopharyngitis (*p* <0.05) and normal nasopharynx tissues (*p* <0.001). Moreover, high level of p-H3Ser10 was positively correlated with the expression of LMP1 in NPC tissues (*χ*^2^=6.700, *p* =0.01; C=0.350) and cell lines. The knockdown and mutant (S10A) of histone H3 suppressed LMP1-induced CNE1 cell proliferation, foci formation and AP-1 activation. In addition, LMP1 could increase MSK1 kinase activity and phosphorylation. MSK1 inhibitor H89 or knockdown of MSK1 by siRNA blocked LMP1-induced phosphorylation of histone H3 at Ser10 and AP-1 activation.

**Conclusion:**

EBV-LMP1 can induce phosphorylation of histone H3 at Ser10 via MSK1. Increased phosphorylation of histone H3 at Ser10 is likely a crucial regulatory mechanism involved in LMP1-induced carcinogenesis of NPC.

## Background

Posttranslational modifications of histone, such as methylation, acetylation, phosphorylation and ubiquitination, are known to play an important role in modulating chromatin structure and regulating gene expression [1]. Phosphorylation of histone H3 at Ser10 is crucial for chromosome condensation and traditionally regarded as a marker of mitosis [2]. Conversely, phosphorylation of histone H3 at Ser10 was observed in interphase after cell stimulation with growth factor, stresses and chemical compounds, and associated with the transcriptional activation of immediate-early (IE) genes, including proto-oncogenes *c*-*fos* and *c*-*jun*[3,4]. The IE gene response has been implicated in proliferation, differentiation and diseases, such as inflammation and cancer [5]. Constitutive activation of Ras-mitogen-activated protein kinase (MAPK) pathway in oncogene-transformed (e.g. H-*ras*) mouse fibroblasts elevated the level of phosphorylated histone H3 at Ser10, accompanying with the aberrant expression of c-*fos*, c-*myc* and uPA gene [6,7]. However, much less is known about the role of histone H3 phosphorylation at Ser10 in neoplastic cell transformation and carcinogenesis.

Accumulating evidences have demonstrated that phosphorylation of histone H3 at Ser10 is involved in different signaling pathways depending on specific stimulation and stress. Fibroblasts with mutations in ribosomal subunit protein S6 kinase 2 (RSK2) gene failed to exhibit epidermal growth factor (EGF)-stimulated phosphorylation of histone H3 at Ser10, suggested that RSK2 is required for EGF-induced phosphorylation of histone H3 [8]. Mitogen- and stress-activated kinase (MSK1) has been shown to mediate EGF, 12-O-tetradecanoyl phorbol-13-acetate (TPA), ultraviolet and oncogene-induced phosphorylation of histone H3 at Ser10 [9-11]. Our previous studies indicated that RSK2, but not MSK1, was involved in arsenite-induced phosphorylation of H3 at Ser10 [12]. All these studies showed that various stimuli probably trigger different kinases to phosphorylate histone H3, thus, it’s very important to identify the responsible kinases and the circumstances mediated histone H3 phosphorylation.

Nasopharyngeal carcinoma (NPC) is a most common malignant tumor in southern China and some regions in Southeast Asia. Its occurrence involves the interaction of host genetic alterations with environmental factors, especially infection by Epstein-Barr virus (EBV) [13]. EBV-encode latent membrane protein 1 (LMP1) is the only latent gene product with transformation properties. It has been shown that LMP1 is crucial for EBV-induced transformation and immortalization of B lymphocytes [14]. Similar oncogenic properties were displayed in rodent fibroblasts and transgenic mice [15]. When expressed in tumorigenic epithelial cells, LMP1 potentiated anchorage-independent growth and greatly promoted migration and invasion [16-18]. Many of the oncogenic effects of LMP1 are attributed to constitutively triggering a plethora of signaling pathways including NF-κB, AP-1 and STAT pathways, which regulates the expression of downstream target genes, thereby mediating tumorigenesis of NPC [17-19]. It has been shown that increased phosphorylation of histone H3 at Ser10 may contribute to the aberrant gene expression and promote oncogene-mediated transformation [6,7]. However, there is no evidence whether phosphorylation of histone H3 at Ser10 is involved in LMP1-induced cell transformation in NPC.

In this study, the expression of histone H3 phosphorylation at ser10 and its correlation with EBV-LMP1 expression in NPC are investigated. Then, we further explore the role of histone H3 phosphorylation at Ser10 in LMP1-induced CNE1 cell transformation and its regulatory kinase.

## Methods

### Patients and tissue specimens

Nasopharyngeal carcinoma tissue microarray (catalog No. NPC961) was from US Biomax (Rockville, MD), including 33 cases of poorly differentiated NPC tissues, 26 cases of adjacent normal tissues, and 10 cases of normal nasopharyngeal tissues. In addition, 15 cases of poorly differentiated NPC tissues and 15 cases of chronic nasopharyngitis tissues were obtained from the First Affiliated Hospital of Guangdong Medical College, Zhanjiang, China. The patients were not pretreated with radiotherapy or chemotherapy prior to surgery. All cases were confirmed by pathological examination and staging was performed according to the 1997 NPC staging system of the WHO. In the 48 NPC cases, there were 37 male and 11 female with age ranging from 26 to 62 years (median, 43.6 years). For the use of these clinical materials for research purposes, prior consent of the patients and approval from the Institutional Ethics Committee of Guangdong Medical College were obtained.

### Cell culture and plasmids

CNE1 cells, an EBV-negative cell line derived from a well-differentiated Chinese NPC patient, were cultured in RPMI 1640 medium supplemented with 10% fetal bovine serum (GIBCO-Invitrogen, Carlsbad, CA) and antibiotics (100 U/ml penicillin,100 g/ml streptomycin). CNE1G (CNE1 stably transformed with PAT-GFP) and CNE1GL(CNE1 stably transfected with PAT-GFP-LMP1) cells were provided by Dr. Xiaoyi Chen, Guangdong Medical College [20], and were maintained in completed RPMI 1640 medium described above, containing 0.5 μg/ml puromycin (Sigma-Aldrich, St. Louis, MO). The pcDNA3.0 and pcDNA3.0-LMP1 vectors were kindly provide by Dr Ellen Cahir-McFarland, Brigham and Women’s Hospital, Boston, Massachusetts, USA. The mU6pro vector was provided by Dr. Zigang Dong, Hormel Institute, University of Minnesota, Austin, Minnesota, USA. The AP-1 reporter vector pRTU14 was kindly provided by Dr ArndKieser, Helmholtz ZentrumMünchen, Munich, Germany [21].

A cDNA fragment encoding human histone H3 was inserted in-frame into the XbaI/EcoRI sites of the pcDNA6.0/myc-His B vector (Invitrogen, Carlsbad, CA) to produce the myc and His epitope-tagged construct, pcDNA6.0-H3. The vector of histone H3 S10A mutant was generated by replacing Ser10 of histone H3 with alanine using the KOD-Plus-Mutagenesis kit (Toyobo Co., Ltd, Osaka, Japan), and named as pcDNA6.0-H3S10A. To construct the siRNA-H3 (si-H3) or siRNA-MSK1 (si-MSK1), the mU6pro vector was digested with XbaI and BbsI. The annealed synthetic primers (H3 siRNA sense: 5^′^-TTTGCAGACAGCTCGGAAATCCATTCAAGAGATGGATTTCCGAGCTGTCTGTTTTTT-3^′^ and antisense: 5^′^-CTAGAAAAAACAGACAGCTCGGAAATCCATCTCTTGAATGGATTTCCGAGCTGTCTG-3^′^; MSK1 siRNA sense: 5^′^-TTTGAGACCTAATTCAGCGTCTTTTCAAGAGAAAGACGCTGAATTAGGTCTTTTTT-3^′^ and antisense: 5^′^-CTAGAAAAAAGACCTAATTCAGCGTCTTTCTCTTGAAAAGACGCTGAATTAGGTCT-3^′^) were then introduced into the mU6pro vector. The recombinant plasmids were confirmed by agarose gel electrophoresis and DNA sequencing.

### Antibodies and reagents

Antibodies against phosphorylated or total histone H3, phosphorylated or total ERK1/2 and MSK1 were purchased from Cell Signaling Technology (Beverly, MA). Anti-EBV LMP1 (CS1-4) antibody was purchased from DAKO (Glostrup, Denmark). Infrared-dye-conjugated secondary antibodies were purchased from Rockland Immunochemicals (Gilbertsville, PA). PD98059 and H89 were purchased from Cell Signaling Technology (Beverly, MA). Pure histone H3 was from NEB (Beverly, Mass). JetPEI transfection reagent was from Polyplus (llkirch, France).

### Immunohistochemistry analysis

Formalin-fixed and paraffin-embedded (FFPE) specimens were cut into 4-μm sections, mounted onto the polylysine-coated slides, deparaffinized in xylene, and rehydrated in a graded ethanol series. Heat-mediated antigen retrieval was performed with sodium citrate buffer (0.01M, pH 6.0). Endogenous peroxidase activity and non-specific antigen were blocked with 3% hydrogen peroxide and normal goat serum. The sections were incubated with the primary antibodies against LMP1 or phosphorylated histone H3 (Ser10) overnight at 4°C. HRP-conjugated secondary antibodies (ChemMate Envision Detection Kit, Dako) were applied onto the sections and incubated for 30 min at room temperature. 10% normal goat serum was used to replace primary antibodies as a negative control. Staining of LMP1 appeared on the cell membrane or/and in the cytoplasm. The percentage of stained cells was determined in 3 representative fields contained at least 200 tumor cells. The expressions of LMP1 were then scored as positive (≥10%) and negative (<10%) based on the percentage of stained cells [22]. The immunoreactivity to histone H3 phosphorylation was localized in the cell nucleus. The number of nuclear stained cells was determined by the examination of at least 1000 cells in 3 representative fields, named as positive labeling index (PLI) for histone H3 phosphorylation [23].

In order to detect the expression of LMP1 and histone H3 phosphorylation at Ser10 in CNE1G and CNE1GL cells, they were immunohistochemically stained using the same staining method as for the clinical specimens.

### Protein extraction and western blot analysis

Extraction of histone protein was performed as described previously [24]. In brief, approximately 1×10^6^ cells were resuspended in 1 mL lysis buffer [10 mmol/L HEPES (pH 7.9), 10 mmol/L KCl, 1.5 mmol/L MgCl_2_, 0.65% NP40, 0.5mmol/L DTT, 1.5mmol/L PMSF]. The lysates were centrifuged at 10000×g for 10 minutes to pellet the intact nuclei. The Nuclei were extracted with 0.4 N H_2_SO_4_ and were incubated on a rotator for at least 30 min. Extraction solutions were centrifuged at 10000×g for 10 min, and acid-insoluble pellets were discard. Supernatant fractions were precipitated with 5 volumes of ice-cold acetone for overnight. The acid-soluble protein was dissolved in 100 μl double-distilled H_2_O. As described elsewhere, total protein was extracted with RIPA lysis buffer (Beyotime Ins. Bio, China).

Protein concentration was determined by the bicinchoninic acid (BCA) assay (Pierce, USA). Samples containing equal amount of protein were resolved by SDS-PAGE and transferred to PVDF membranes (millipore, Billerica, MA). The membranes were blocked with 5% not-fat dried milk for 2 hours, and then probed with the primary antibodies overnight at 4°C. After washing with 0.1% Tween-20 in TBS, membranes were incubated with infrared-dye-conjugated secondary antibodies for 1 hour at room temperature. Protein bands were visualized by Odyssey Infrared Imaging System (LI-COR Biotechnology, Lincoln, NE, USA).

### Cell counting kit-8 (CCK-8) assay

The cell proliferative ability was evaluated by CCK-8 (Dojindo Laboratories, Kumamoto, Japan) assay. CNE1G or CNE1GL cells were transfected with si-mock or si-H3 plasmids and then seeded in 96-well plates (3×10^3^ per well). After culturing for various periods of time, CCK-8 solution (10 μl per 100 μl medium) was added to each well, and cells were then incubated for 1 hour at 37°C. Absorbance was measured at 450 nm using Synergy2 Multi-Mode Microplate Reader (BioTek, Winooski, VT, USA). The assay was conducted in five replicate wells for each sample and three parallel experiments were performed.

### Focus-forming assay

The transformation potential of the introduced genes in cells was evaluated by Focus-forming assay. CNE1 cells were transiently transfected with various combinations of expression vectors and seeded in six-well plates (500 per well). After culturing for 2 weeks, foci were fixed with methanol and stained with 0.5% crystal violet. Foci containing more than 50 cells were considered, and the mean values from three replicate wells were calculated. Data are representative of at least three independent experiments.

### Reporter gene assay

Activator protein-1 (AP-1) activation was determined by the luciferase reporter gene assay. Cells were transiently cotransfected with AP-1 reporter gene and pRL-TK vector (Promega, China). The pRL-TK vector expressing *Renilla* luciferase was cotransfected to calibrate the firefly luciferase activity. Cells were lysed with passive lysis buffer (Promega) for 20 min with gently shaking. Luciferase activities were measured with cell lysates using the Dual-Luciferase assay system (Promega) in FB12 Luminometer (Berthold detection system). The firefly luciferase activity was normalized against *Renilla* luciferase activity. Data were derived from the mean of triplicate samples and recorded as relative luciferase activity (fold or %). All experiments were done at least in triplicate.

### Histone H3 Kinase Assay *in vitro*

Cell extracts (20 μg) of CNE1G and CNE1GL cells were incubated in 1×kinase buffer supplemented with 1 μg of pure histone H3, 200 μM ATP, and presence or absence of 10 μM H89 for 30 min at 30°C. Reactions were terminated with 6×SDS sample buffer. The samples were denatured at 95–100°C for 5 min before they were separated by 15% SDS-PAGE. The phosphorylation of histone H3 at Ser10 and total histone H3 protein were detected by western blot with specific antibodies.

### MSK1 kinase assay *in vitro*

Cell extracts (200 μg) of CNE1G and CNE1GL cells were incubated with immobilized Phospho-MSK1 (Thr581) monoclonal antibody overnight at 4°C. Then protein A/G agarose beads (20 μl) were added and incubated for 2 hours at 4°C. These samples were washed three times with 500 μl of 1×cell lysis buffer, and then washed twice with 500 μl of 1×kinase buffer. The pellets were suspended in 40 μl of 1×kinase buffer supplemented with 1 μg of histone H3 protein and 200 μM ATP, and incubated for 30 min at 30°C. Reactions were terminated with 6×SDS sample buffer, and then samples were separated by 15% SDS-PAGE. MSK1 kinase activity for histone H3 was analyzed by western blot using anti-phosphorylated histone H3 antibody.

### Statistical analysis

Quantitative values were expressed as means ± SD. The SPSS version 16.0 software package and GraphPad Prism were used for the statistical analysis and data plotting. Student *t*-test was used to compare the mean value of each group. The relationship between LMP1 and histone H3 phosphorylation expression was analyzed using Chi-square test. *p*<0.05 was considered statistically significant.

## Results

### Expression of histone H3 phosphorylation at Ser10 and its correlation with LMP1 in NPC tissues

**Figure 1:**
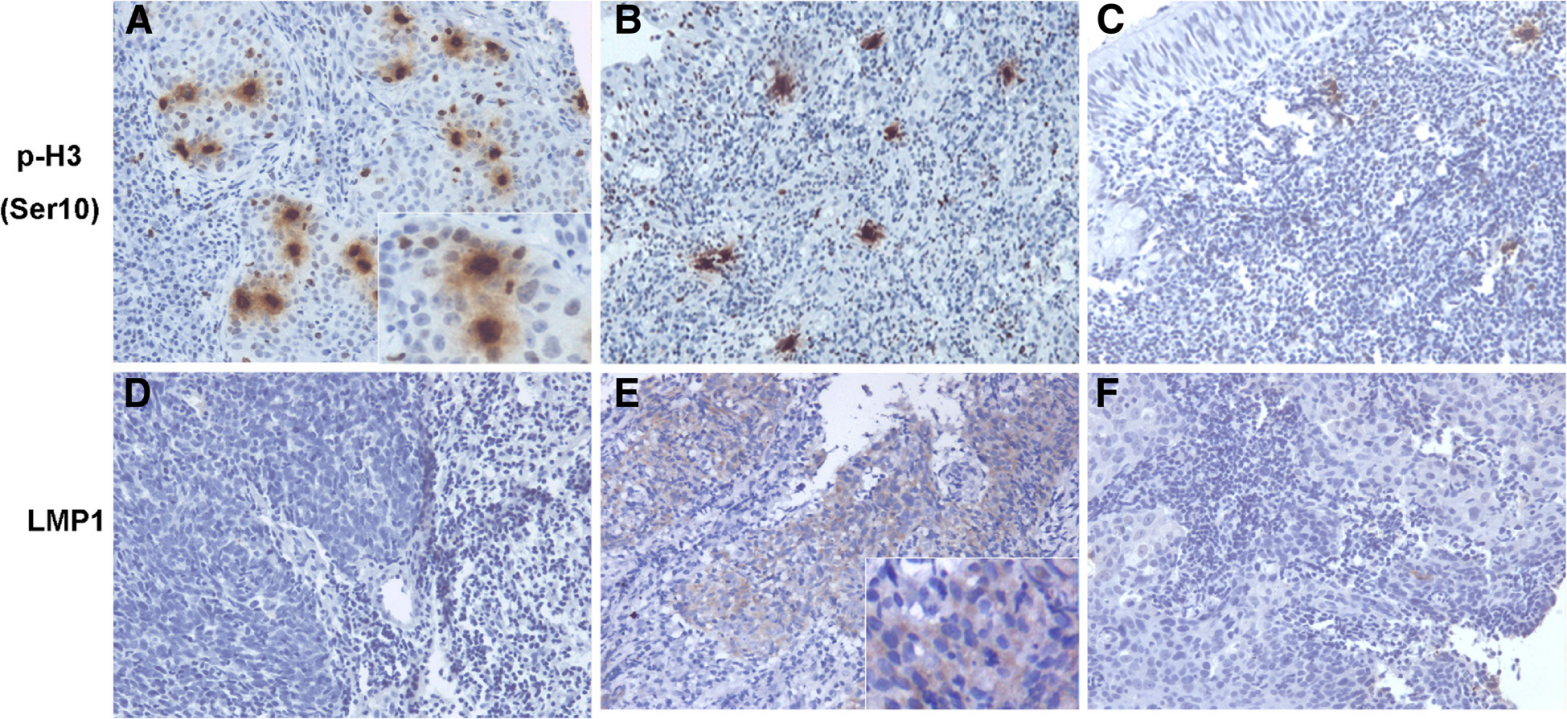
**Expression of histone H3 phosphorylation at Ser10 and LMP1 in NPC samples.** Different expression of histone H3 phosphorylation at Ser10 in poorly differentiated NPC (**A**), chronic nasopharyngitis (**B**) and nomal nasopharynx tissues (**C**). Negative expression (**D**) and positive expression (**E**) of LMP1 in NPC tissues. Example incubated with 10% normal goat serum as a negative control (**F**). Images were captured at ×200 magnification, and ×400 magnification for inserts.

In order to assess the role of histone H3 phosphorylation at Ser10 in the tumorigenesis of NPC, we analyzed the expression level of histone H3 phosphorylation in 48 archived paraffin-embedded NPC specimens, 15 chronic nasopharyngitis specimens and 36 adjacent/normal nasopharynx specimens using immunohistochemical staining. The phosphorylation of histone H3 at Ser10 was diffusely expressed in cell nuclei (Figure 1A-C). As shown in Figure 1 and Table 1, p-H3Ser10 positive labeling index was significantly higher in the poorly differentiated NPC tissues than that in chronic nasopharyngitis tissues (*p*<0.05) and normal nasopharynx tissues (*p*<0.001). Moreover, we found that the expression level of histone H3 phosphorylation was higher in chronic nasopharyngitis, compared with normal nasopharynx tissues (*p*<0.005). This revealed that the increased phosphorylation of histone H3 at Ser10 may involve in the malignant transformation of NPC cells.

**Table 1:** **The positive labeling index** (**PLI**) **for histone H3 phosphorylation at Ser10 in nasopharynx samples**

**Groups**	**N**	**p-H3Ser10 labeling index (mean±SD)**
poorly differentiated NPC	48	22.46±10.59 ^*^
chronic nasopharyngitis	15	14.87±6.06 ^#^
normal nasopharynx	36	8.27±4.65

*Compared with chronic nasopharyngitis (*p* < 0.05) or normal nasopharynx tissues (*p* < 0.001). ^#^ Compared with normal nasopharynx tissues (*p* < 0.005).

We further determined the relationship between histone H3 phosphorylation at Ser10 and LMP1 expression in 48 cases of NPC specimens. The LMP1 expression was located on cell membrane and cytoplasm (Figure 1E). In NPC, 28 out of 48 (58.3%) cases showed LMP1 expression. For statistical analysis, the expression levels of p-H3Ser10 were classified into low and high labeling index groups according to the mean of labeling index. As shown in Table 2, there was a positive correlation between LMP1 expression and abnormal expression of histone H3 phosphorylation at Ser10 in NPC tissues (*χ*^2^=6.700, *p* =0.01; C=0.350).

**Table 2:** The relationship between LMP1 expression and expression of histone H3 phosphorylation at Ser10 in NPC tissues

**Groups**		**LMP1**	
		**+**	**-**
H3-P labeling index	high	19(39.6%)	6(12.5%)
	low	9(18.8%)	14(29.2%)

*χ*^2^=6.700, *p* =0.01; contingency coefficient: C=0.350.

### LMP1 induced phosphorylation of histone H3 at Ser10 in CNE1 cells

**Figure 2:**
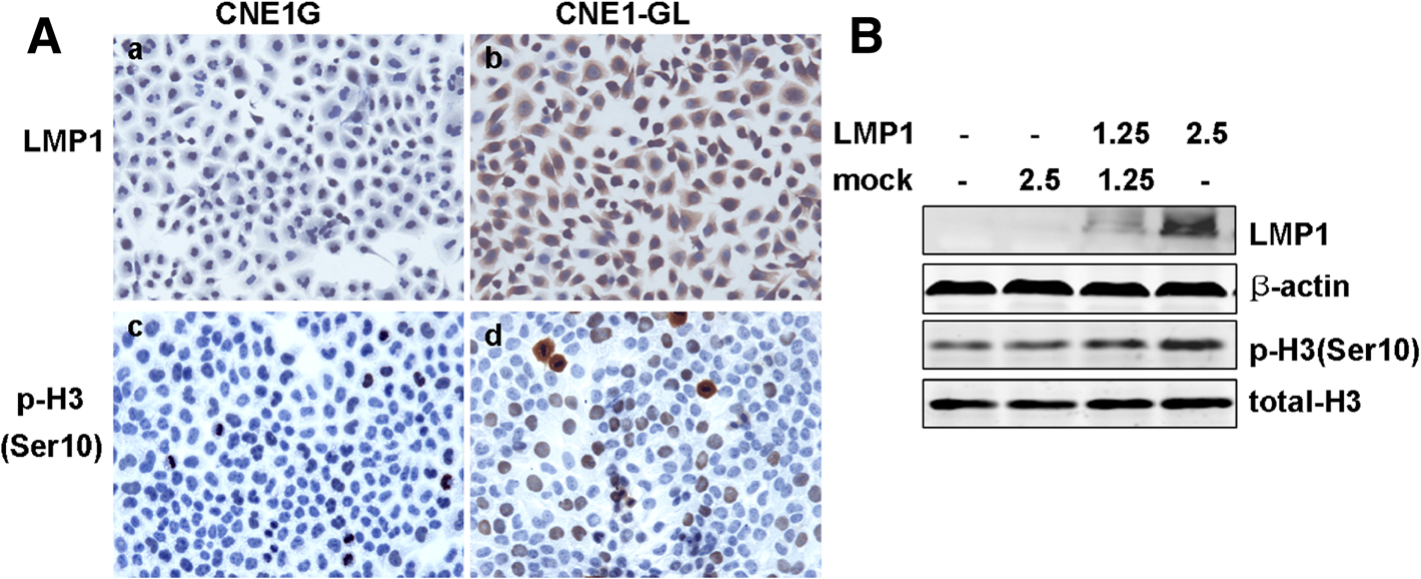
**LMP1 induced phosphorylation of histone H3 at Ser10 in CNE1 cells.** (**A**) CNE1G and CNE1GL cells were serum-starved for 36 h and subjected to immunocytochemical staining (×200). No expression and positive staining of LMP1 in CNE1G and CNE1GL cells (a, b). Different expression of histone H3 phosphorylation at Ser10 in CNE1G and CNE1GL cells (c, d). (**B**) CNE1 cells were transfected with different amount of pcDNA3.0 (1.25-2.5 μg) or pcDNA3.0-LMP1 (1.25-2.5 μg). After 24h of transfection, cells were starved for another 24 h. Total protein and histone protein were extracted and the expressions of LMP1 and phosphorylated histone H3 were detected by Western blot analysis. β-actin and total histone H3 were used as loading controls.

To investigate whether LMP1 induced phosphorylation of histone H3 at Ser10 in NPC cells, we examined the relative levels of phosphorylated histone H3 at Ser10 between CNE1G and CNE1GL cells by immunocytochemical staining. In serum-starved CNE1G cells, the expressions of phosphorylated histone H3 were observed mainly in cells in mitotic phase. In contrast, CNE1GL cells exhibited more extensive expressions although they showed different stain pattern. Most of them showed nuclear dot-like staining (Figure 2A). In addition, CNE1 were transiently transfected with different amount of pcDNA3.0-LMP1 or pcDNA3.0 (compensation to achieve equal amount DNA), then the expression level of LMP1 and phosphorylation of histone H3 at Ser10 were examined by western blot analysis. As shown in Figure 2B, phosphorylation of histone H3 at Ser10 was increased in a dose-dependent manner with the expression of LMP1. Similar change was also observed in LMP1-transfected CNE2 cells, a poorly-differentiated NPC cell line (Additional file 1). These results indicated EBV-LMP1 could constitutively activate the phosphorylation of histone H3 at Ser10 in NPC cells.

### Phosphorylation of histone H3 at Ser10 was involved in LMP1-induced CNE1 cell transformation

**Figure 3:**
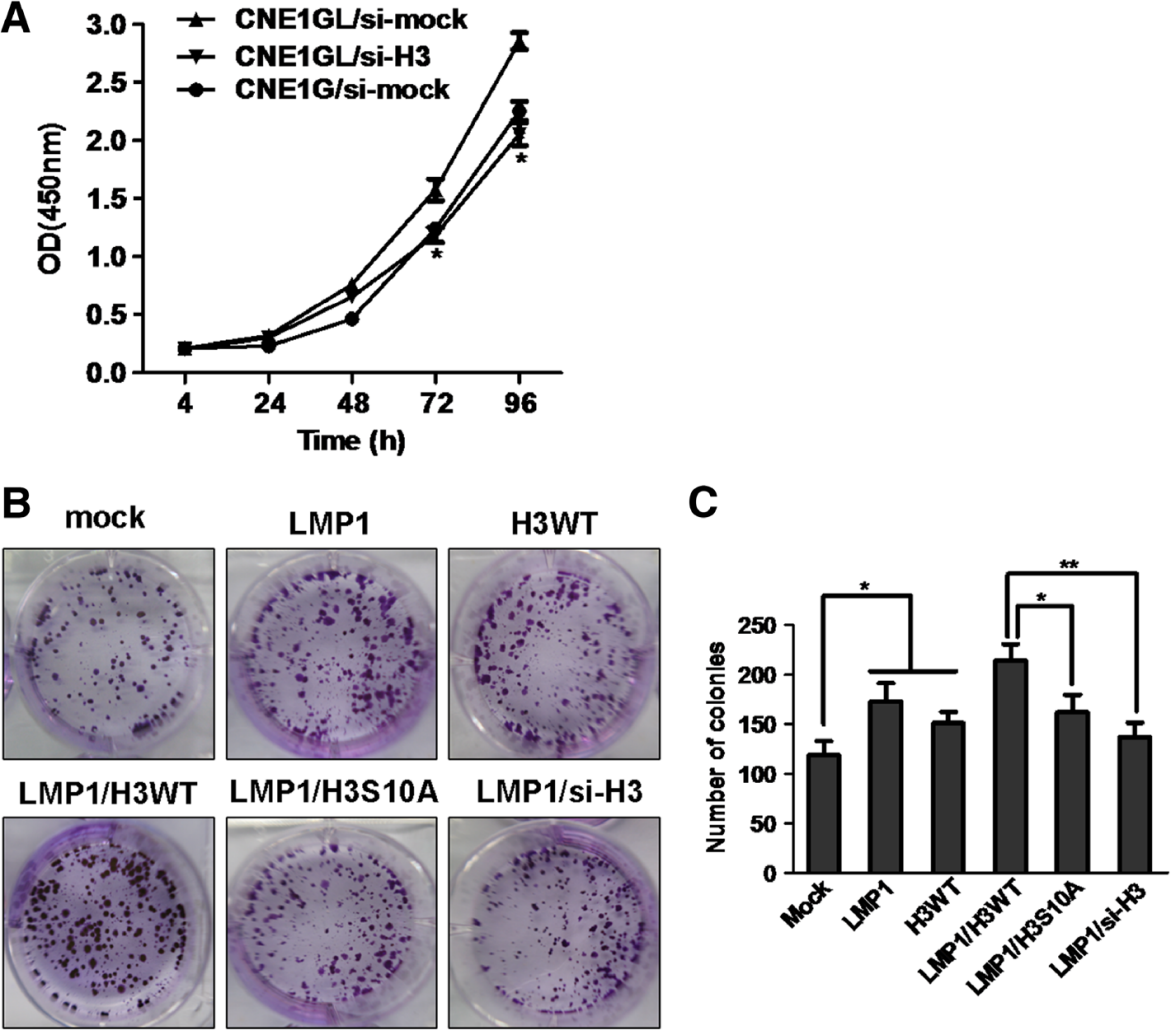
**Phosphorylation of histone H3 at Ser10 was involved in LMP1**-**induced CNE1 cell transformation.** (**A**) CNE1G and CNE1GL cells were transfected with si-mock or si-H3 and then cell proliferation was estimated at 24-hour intervals up to 96 hours using CCK-8 assay. Data were presented using mean±SD. Asterisks indicate a significant difference compared with CNE1GL/si-mock control cells (*, *p* < 0.01). (**B**) **and** (**C**) CNE1 cells transfected with various combinations of expression vectors as indicated were subjected to a focus-forming assay. The cells were cultured for 2 weeks and foci were stained with 0.5% crystal violet. The average foci number was calculated and presented using mean±SD. Asterisks indicate a significant difference in foci formation between indicated groups. (*, *p* < 0.05; **, *p* < 0.005).

It has been shown that LMP1 induced the phosphorylation of histone H3 at Ser10 in CNE1 cells. We next explored whether histone H3 phosphorylation at Ser10 is crucial for cell transformation exerted by LMP1. We designed siRNA against histone H3 (si-H3) and a scrambled control siRNA (si-mock) for transfecting into CNE1GL cells. Quantitative RT-PCR (Additional file 2) and immunoblot analysis revealed that the si-H3 could effectively down-regulate the expression of endogenous histone H3 (Additional file 3). After being transfected by si-mock or si-H3, cell proliferation was analyzed by CCK-8 assay. The results indicated that knockdown of histone H3 in CNE1GL cells (CNE1GL/si-H3) markedly suppressed cell proliferation compared with the si-mock control cells (CNE1GL/si-mock; Figure 3A). Notably, LMP1 stable CNE1 cells transfected with si-mock (CNE1GL/si-mock) showed an increase in cell proliferation compared with mock stable cells (CNE1G/si-mock; Figure 3A). The results suggested that histone H3 was involved in CNE1 cell proliferation promoted by LMP1.

To further study whether the histone H3 phosphorylatable motif at Ser10 specifically regulated cell transformation promoted by LMP1, we replaced Ser10 of histone H3 with alanine by site-mutagenesis to generate the mutant histone H3 expression vector (H3 S10A). Expressions of vectors were confirmed with an antibody against the His epitope (Additional file 3). Various combination of the expression vectors were cotransfected into CNE1 cells, and then the effects on foci formation were analyzed. Our results showed that LMP1 or histone H3 overexpression promoted an increase of transformation foci in CNE1 cells (Figure 3B, top middle, right and Figure 3C). Importantly, coexpression of LMP1 and H3 WT promoted more foci formation compared with transfection of LMP1 and H3 S10A mutant (Figure 3B, bottom left, middle and Figure 3C). Moreover, cotransfection of LMP1 with si-H3 effectively blocked foci formation in CNE1 cells (Figure 3B, bottom right and Figure 3C). These results indicated that the phosphorylation of histone H3 at Ser10 was most likely a critical site for regulating LMP1-induced CNE1 cells transformation.

### MSK1 mediated LMP1-induced phosphorylation of histone H3 at Ser10 in CNE1 cells

**Figure 4:**
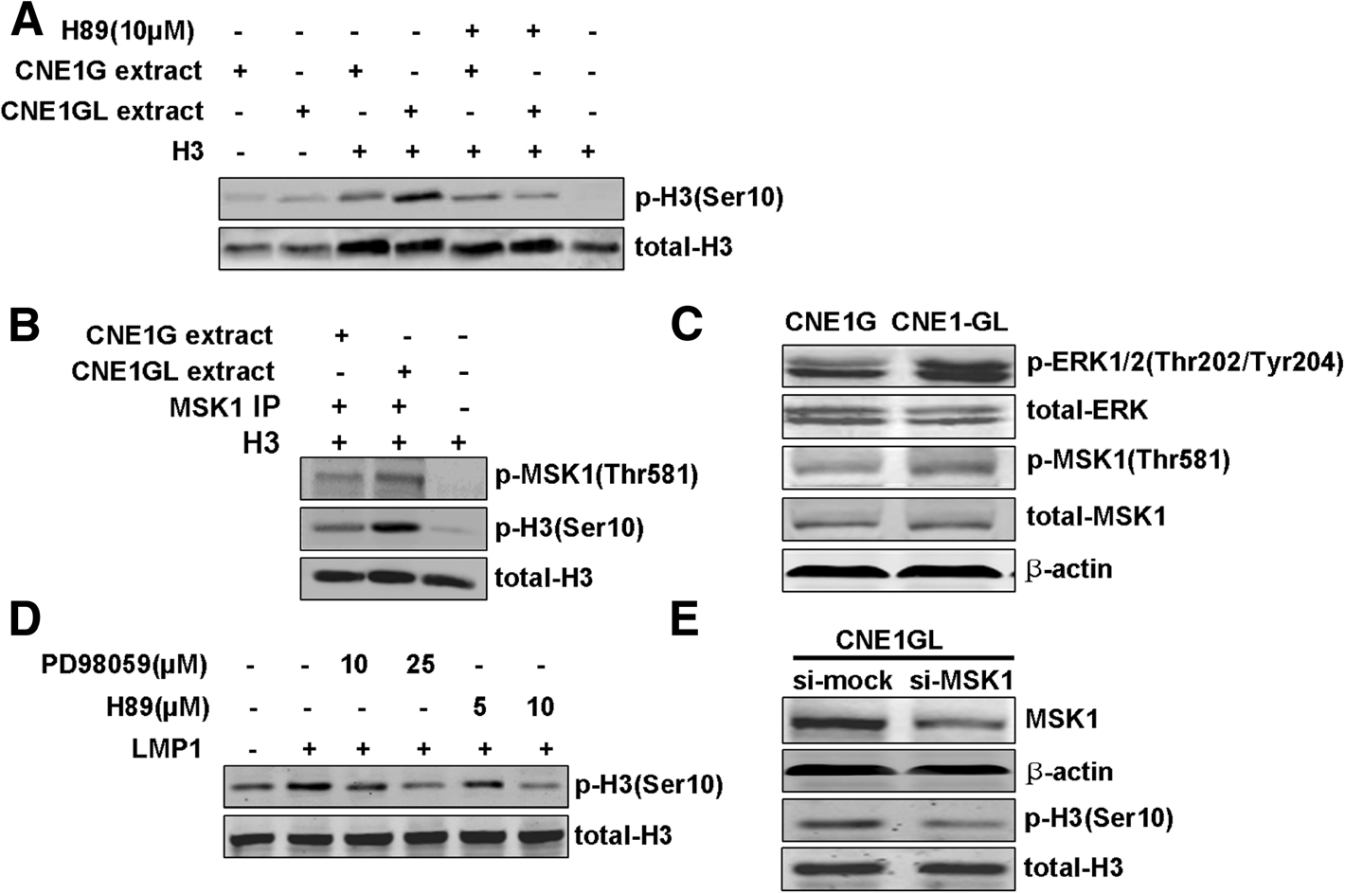
**MSK1 activity was required for LMP1**-**induced phosphorylation of histone H3 at Ser10.** (**A**) Histone H3 kinase activity in CNE1G and CNE1GL cells. Cell extracts (20 μg) were incubated with the pure histone H3 protein (1 μg), ATP (200 μM), and the presence or absence of H89 (10 μM). The expressions of phosphorylated and total histone H3 were detected by Western blot analysis. (**B**) MSK1 kinase activity in CNE1G and CNE1GL cells. Equal amounts of whole cell extracts (200 μg) were immunoprecipitated with anti-phosphorylated MSK1 antibody, and then incubated with the pure histone H3 protein (1 μg), ATP (200 μM). The expressions of phosphorylated and total histone H3 were detected by Western blot analysis. (**C**) CNE1G and CNE1GL cells were serum-starved for 36h and the expressions of phosphorylated and total ERK1/2 or MSK1 were detected by Western blot analysis. (**D**) CNE1 cells were transfected with pcDNA3.0 or pcDNA3.0-LMP1 vector. PD98059 or H89 was added to the culture medium at the concentration indicated every 12 h after transfection. Histone protein was extracted and the expressions of phosphorylated and total histone H3 were detected by Western blot analysis. **(E)** CNE1GL cells were transfected with si-mock or si-MSK1 vector. After 48 h of transfection, total protein and histone protein were extracted. The expressions of MSK1 and phosphorylated histone H3 were detected by Western blot analysis. β-actin and total histone H3 were used as loading controls.

To explore the signaling mechanism for histone H3 phosphorylation at Ser10, we examined histone H3 kinase activity in serum-starved CNE1G and CNE1GL cells. *In vitro* H3 kinase assays with equal amount of cell extracted protein, our results showed that H3 kinase activity in the LMP1-transfected CNE1 cells was greater than that from the mock control cells in the presence of histone H3 substrate (Figure 4A). However, pretreatment of H89 significantly decreased the H3 kinase activity in both cell extracts (Figure 4A). The remaining H3 kinase activity may be Aurora B, the mitotic H3 kinase. To directly test whether LMP1 increased the MSK1 kinase activity, MSK1 was immunoprecipitated from the cell extracts isolated from CNE1G and CNE1GL cells with anti-phospho-MSK1 (Thr581), and then MSK1 kinase activity was assayed *in vitro* with histone H3 as a substrate. The results showed that LMP1 greatly increased MSK1 kinase activity for histone H3 (Figure 4B).

The phosphorylation levels of ERK1/2 and MSK1 were detected by western blot analysis. Our results showed that LMP1 obviously activated the phosphorylation of ERK1/2 and MSK1 in CNE1 cells (Figure 4C). ERK1/2 inhibitor PD98059 and MSK1 inhibitor H89 were used to treat the LMP1-transfected CNE1 cells. We found that a relatively low concentration PD98059 (10 μM) and H89 (5 μM) inhibited the phosphorylation of histone H3 at Ser10 in a dose-dependent manner (Figure 4D). In addition, we designed siRNA against MSK1 (si-MSK1) for transfecting into CNE1GL cells. The results from quantitative RT-PCR (Additional file 2) and western blot showed that the expression of MSK1 was markedly decreased in si-MSK1 transfected cells (Additional file 3 and Figure 4E). Consistent to the effect of treatment with H89, the knockdown of MSK1 by siRNA also resulted in a loss of histone H3 phosphorylation at Ser10 in CNE1GL cells (Figure 4E). These results indicated that Ras-MAPK pathway and MSK1 might mediate LMP1-induced phosphorylation of histone H3 at Ser10 in CNE1 cells.

### MSK1-mediated histone H3 phosphorylation at Ser10 regulated LMP1-induced AP-1 activation in CNE1 cells

**Figure 5:**
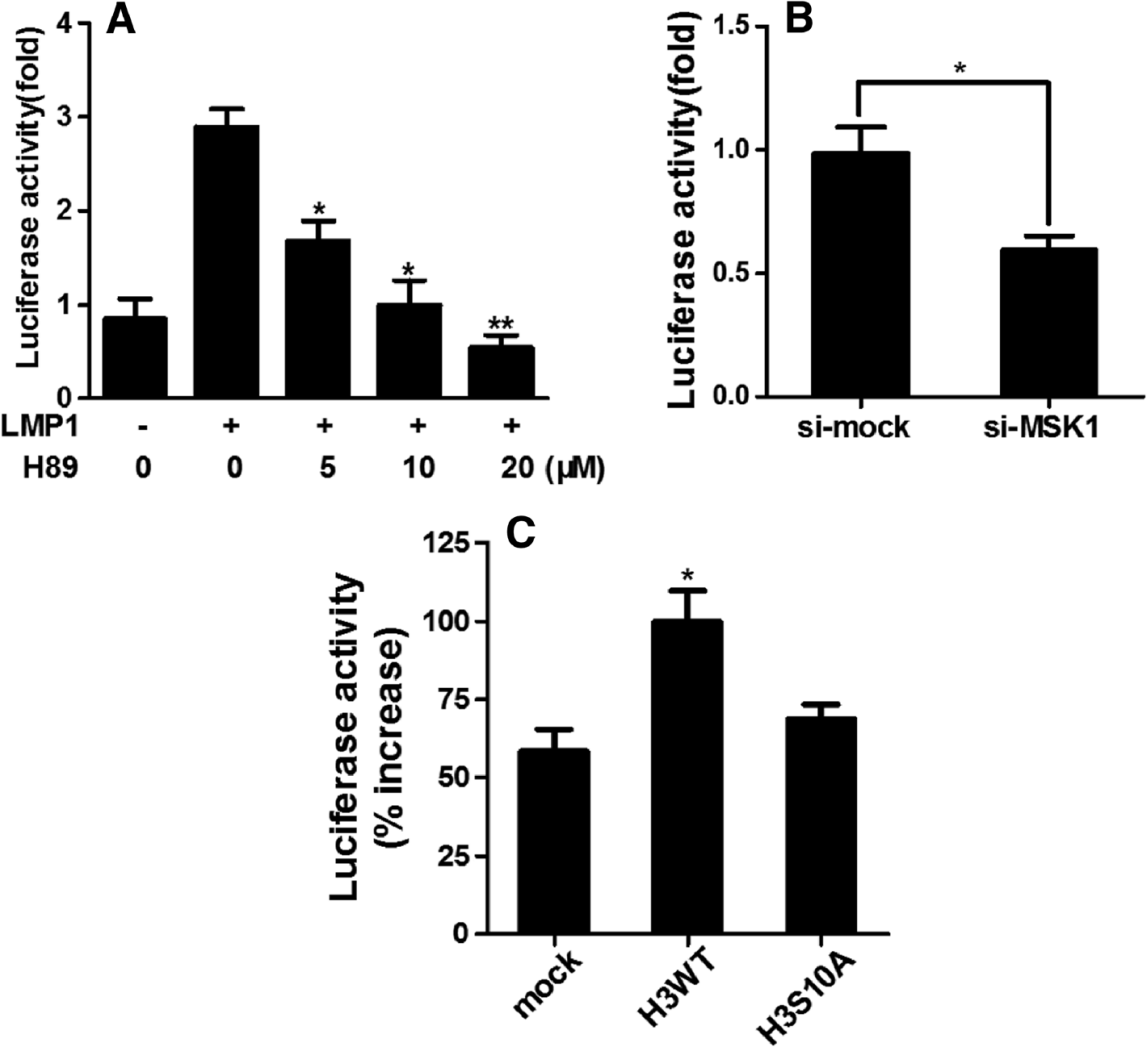
**MSK1**-**mediated histone H3 phosphorylation at Ser10 regulated LMP1**-**induced AP**-**1 activation in CNE1 cells.** (**A**) CNE1 cells were transfected with pcDNA3.0 or pcDNA3.0-LMP1. H89 was added to the culture medium at the concentration indicated every 12 h after transfection. The AP-1 firefly luciferase activity was measured and normalized against *Renilla* luciferase activity. Asterisks indicate a significant difference compared with LMP1-transfected cells untreated with H89 (*, *p* < 0.05; **, *p* < 0.005). (**B**) CNE1 cells were cotransfected with si-mock or si-MSK1 and pcDNA3.0-LMP1. At 48 hours after transfection, the AP-1 firefly luciferase activity was measured and normalized against *Renilla* luciferase activity. Asterisks indicate a significant difference between two groups (*, *p*< 0.05). (**C**) CNE1 cells were cotransfected with pcDNA6.0, pcDNA6.0-H3 or pcDNA6.0-H3S10A and pcDNA3.0-LMP1. After 24 h of transfection, cells were starved for another 24 h. The AP-1 firefly luciferase activity was measured and normalized against *Renilla* luciferase activity. Asterisks indicate a significant difference compared with mock control cells (*, *p*< 0.05).

The AP-1 transcription factor is a heterodimeric protein formed by c-*fos*, c-*jun*, activating transcription factor (ATF) and musculoaponeurotic fibrosarcoma (MAF) protein families [25]. The regulation of cell proliferation by AP-1 is implicated in the malignant transformation [25,26]. Here, we cotransfected the AP-1 reporter plasmid and pcDNA3.0-LMP1 or pcDNA3.0 into CNE1 cells. The results showed that LMP1 increased the AP-1 promoter activity by 3-fold (Figure 5A). However, the treatment of H89 dramatically suppressed the LMP1-promoted AP-1 activation in a dose-dependent manner (Figure 5A). We further tested the effect of MSK1 knockdown on LMP1-promoted AP-1 activation. Consistently, AP-1 activation was suppressed in si-MSK1 transfected cells compared with si-mock control cells (Figure 5B). These results indicated that MSK1 played an important role in regulating LMP1-induced AP-1 activation.

To determined whether histone H3 phosphorylation at Ser10 might directly regulate LMP1-induced AP-1 activation, mock, H3 WT or H3 S10A mutant was cotransfected with AP-1 reporter plasmid into LMP1 expressing CNE1 cells. The LMP1-induced AP-1 activation response was more pronounced in H3 WT-overexpressing cells than in mock control cells (Figure 5C). In contrast, there were no significant gains of AP-1 activation in H3 S10A mutant-overexpressing cells (Figure 5C). Overall, these results indicated that the AP-1 activation promoted by LMP1 might be regulated through MSK1-mediated histone H3 phosphorylation at Ser10.

## Discussion

Phosphorylation of histone H3 at Ser10 is correlated closely with chromosome condensation, mitosis and gene expression. Many tumor promotion agents, such as EGF, TPA, or ultraviolet, and transformation by oncogene H-*ras* or v-Src can elevate the level of phosphorylated histone H3 at Ser10 [6,10,11]. Increased phosphorylation of histone H3 as a result of AIM-1/Aurora B overexpression contributed to chromosome instability and was observed in many tumor cell lines, including colorectal and hepatocellular carcinomas [23,27]. These observations implied that the deregulation of histone H3 phosphorylation may play a role in carcinogenesis. In this study, using immunostaining analysis, we found that the p-H3Ser10 positive index in poorly differentiated NPC was significantly higher than that in chronic nasopharyngitis and normal nasopharynx tissues. It is indicated that the increasing phosphorylation of histone H3 might be an important event in NPC pathogenesis and promoted the malignant transformation of nasopharyngeal epithelium. Compared with normal nasopharynx tissues, chronic nasopharyngitis exhibited a higher level of phosphorylated histone H3 at Ser10. It might be associated with chronic stimulation of the nasopharynx from various factors, such as chemical agents, cigarette smoking and viral or bacterial infection, which were shown to induce the phosphorylation of histone H3 at Ser10 [28-30]. However, the specific mechanism remains to be further studied.

LMP1 is the only EBV-encoded latent gene with classical transforming properties, which is closely associated with the carcinogenesis of NPC [17,18]. LMP1 functions as a viral mimic of tumor necrosis factor receptor (TNFR) family member, CD40, and thus triggers a number of cellular signaling pathways, which participates in regulation of cell proliferation, apoptosis, malignant transformation, invasion and metastasis [31,32]. In this study, we found that the elevated expression level of histone H3 phosphorylation in NPC tissues was closely related to LMP1 expression. Moreover, the phosphorylation of histone H3 at Ser10 was more frequently observed in LMP1-transfected CNE1 cells compared with mock control cells in the serum-starved condition. It was found that the most CNE1GL cells with p-H3Ser10 expression did not belong to the G2/M phase of cell cycle. Similar result was also observed in v-Src-transformation mouse fibroblasts [11]. The findings suggested that EBV-LMP1 can constitutively activate phosphorylation of histone H3 at Ser10 in interphase and may contribute to the aberrant expression of IE genes.

Recent studies showed that histone H3, especially the Ser10 motif, has oncogenic effects and directly regulated EGF- or TPA-induced neoplastic cell transformation and cell proliferation [10,33]. Here, we used the knockdown and mutant of histone H3 to explore the role of histone H3 phosphorylation at Ser10 in regulating LMP1-promoted cell transformation of CNE1 cells. The results showed that the knockdown of histone H3 by siRNA suppressed the LMP1-induced cell proliferation and foci formation. Moreover, we found that overexpression of mutation histone H3 (H3S10A) also inhibited foci formation promoted by LMP1 in CNE1 cells compared with overexpressing H3 WT cells. These observations indicated that the phosphorylation of histone H3 at Ser10 might be a crucial regulatory mechanism for LMP1-induced cell transformation in NPC.

*In vitro* histone H3 kinase assay showed that H3 kinase activity in the LMP1-transfected CNE1 cells was greater than that in the mock control cells. But the presence of H89, an inhibitor of MSK1, significantly reduced the H3 kinase activity. We surmised that increasing MSK1 kinase activity may account for the increasing phosphorylation level of histone H3 at Ser10. MSK1 is a nuclear kinase which is activated by the ERK and p38 MAPKs in response to extracellular stimuli. MSK1 has been shown to activate various transcription factors, including cyclic AMP (cAMP)-response element-binding protein (CREB), ATF1, STAT3 and NF-κB, and alters their target DNA-binding capacity or promotes the recruitment of their coactivators [34-36]. Persistent activation of Ras-MAPK pathway and elevated MSK1 activity were observed in many human cancers and tumor cell lines [37,38]. MSK1 has also been reported to phosphorylate the chromatin protein histone H3 and high mobility group 14 (HMG-14) when induced by mitogen- and stress- stimuli [39]. The Ras-MAPK pathway and MSK1 appear to play a critical role in the phosphorylation of histone H3 and oncogenic growth of v-Src transformed cells [11]. In this study, we found that LMP1 increased the phosphorylation level of MSK1 at Thr581 and enhanced the MSK1 kinase activity. ERK1/2 inhibitor PD98059 and MSK1 inhibitor H89 obviously suppressed LMP1-induced phosphorylation of histone H3 at Ser10. Similar results were obtained with MSK1-specific siRNA. These results strongly suggested that LMP1 induced phosphorylation of histone H3 at Ser10 via activation of Ras-MAPK pathway and MSK1 kinase.

Previous studies suggested the AP-1 signaling pathway played an important role in LMP1-mediated tumorigenesis of NPC [17,18]. LMP1 activated c-Jun N-terminal kinases (JNK) and promoted the formation of c-Jun/JunB heterodimers leading to expression of AP-1 regulated gene [40,41]. In present study, we showed the relationship of MSK1-mediated histone H3 phosphorylation and AP-1 transactivation promoted by LMP1 in CNE1 cells. MSK1 inhibitor H89 or knockdown of MSK1 by siRNA significantly suppressed LMP1-promoted AP-1 activation. Furthermore, histone H3, especially the Ser10 motif, also regulated AP-1 activation promoted by LMP1. It was revealed that c-*jun* or c-*fos* gene was a common target of histone H3 leading to induction of AP-1 activity [33]. The activation of the *c*-*fos* serum responsive element (SRE) by histone H3 phosphorylation might promote c-Fos expression and stabilize the c-Fos/c-Jun heterodimer [42]. The increasing AP-1 transactivation activity coupled with histone H3 phosphorylation may contribute to elucidate the mechanism of neoplastic cell transformation mediated by post-translational modification of histone H3. Take together, these results indicated that histone H3 phosphorylation at Ser10 mediated by MSK1 was required for AP-1 activation promoted by LMP1, which was very much associated with LMP1-induced cell transformation. In addition, MSK1-mediated phosphorylation of transcription factors CREB and ATF1 has been shown to induce *c*-*fos* and *junB* transcription [35], and thereby might regulate AP-1 transactivation.

## Conclusion

In summary, this study demonstrated that the level of histone H3 phosphorylation at Ser10 was significantly increased in NPC and positively correlated with the expression of EBV-LMP1. We found that LMP1 induced phosphorylation of histone H3 at Ser10 through the activation of Ras-MAPK pathway and MSK1 kinase in CNE1 cells. Moreover, phosphorylation of histone H3 at Ser10 might play a regulatory role for LMP1-induced cell transformation and AP-1 transactivation. These findings provided new insight into understanding the epigenetic mechanism involved in LMP1 carcinogenesis of NPC. Histone H3 may consider as a crucial target of diagnosis and therapy in the future.

## Abbreviations

NPC: nasopharyngeal carcinoma; EBV: Epstein-Barr virus; LMP1: latent memberane protein 1; MSK1: mitogen- and stress-activated kinase 1; MAPK: mitogen-activated protein kinase; AP-1: activator protein-1

## Competing interests

The authors declare that they have no competing interests.

## Authors’ contributions

BB L designed and performed the experiments, analyzed data and drafted the manuscript. G H and X Z contributed reagents, performed experiments and analyzed data. R L and J W performed experiments and analyzed data. Z D and Z H designed the experiments, analyzed data and drafted the manuscript. All authors read and approved the final manuscript.

## Pre-publication history

The pre-publication history for this paper can be accessed here:

http://www.biomedcentral.com/1471-2407/13/124/prepub

## Supplementary Material

Additional file 1 LMP1 induced phosphorylation of histone H3 at Ser10 in CNE2 cells. Click here for file

Additional file 2 Materials and methods for extraction of total RNA and RT-PCR. Click here for file

Additional file 3 The expressions of various genes were detected after transfection in CNE1GL and CNE1 cells by qRT-PCR and western blot analysis. Click here for file
