# Supplementary material for: Increased phosphorylation of histone H3 at serine 10 is involved in Epstein-Barr virus latent membrane protein-1-induced carcinogenesis of nasopharyngeal carcinoma
Source: BMC Cancer. 2013 Mar 18;13:124. doi: 10.1186/1471-2407-13-124 (PMC3610199; doi:10.1186/1471-2407-13-124)
Supplement: Additional file 1 — LMP1 induced phosphorylation of histone H3 at Ser10 in CNE2 cells. [file 1471-2407-13-124-S1.doc]

**Additional file 1**

Fig.1. LMP1 induced phosphorylation of histone H3 at Ser10 in CNE2 cells. CNE2 cells were transfected with pcDNA3.0 or pcDNA3.0-LMP1. After 24h of transfection, cells were starved for another 36h. Total protein and histone protein were extracted and the expressions of LMP1 and phosphorylated histone H3 were detected by Western blot analysis. β-actin and total histone H3 were used as loading controls.
