# Supplementary material for: Increased phosphorylation of histone H3 at serine 10 is involved in Epstein-Barr virus latent membrane protein-1-induced carcinogenesis of nasopharyngeal carcinoma
Source: BMC Cancer. 2013 Mar 18;13:124. doi: 10.1186/1471-2407-13-124 (PMC3610199; doi:10.1186/1471-2407-13-124)
Supplement: Additional file 2 — Materials and methods for extraction of total RNA and RT-PCR. [file 1471-2407-13-124-S2.doc]

**Additional file 3**

**Materials and methods**

**Extraction of total RNA and RT-PCR**

Total RNA was extracted from cells with TRIzol (Invitrogen, Carlsbad, CA) and cDNA was synthesized by using First Strand cDNA Synthesis kit (Roche, Indianapolis, IN). Real-time quantitative PCR was performed to measure the mRNA expressions of histone H3 and MSK1 in CNE1GL cells using FS Universal SYBR Green Master (Roche) with ABI 7500 real-time PCR system. Primer sequences for histone H3: forward 5'- GTTGCTGATTCGGAAGCTGC -3' and reverse 5'- GAAGCGAAGATCGGTCTTGAA -3'; for MSK1: forward 5'- AGGCAGTCGCCATTTTTGGTA -3' and reverse 5'- TCTCCAACA

TAAATCTGCACCTC -3'; for GAPDH: forward 5'-CTCCTCCTGTTCGACAGTCAGC-3' and reverse 5'- CCCAATACGACCAAATCCGTT -3'. The experiments were repeated three times, and the mean fold changes were recorded.
