# Supplementary material for: Increased phosphorylation of histone H3 at serine 10 is involved in Epstein-Barr virus latent membrane protein-1-induced carcinogenesis of nasopharyngeal carcinoma
Source: BMC Cancer. 2013 Mar 18;13:124. doi: 10.1186/1471-2407-13-124 (PMC3610199; doi:10.1186/1471-2407-13-124)
Supplement: Additional file 3 — The expressions of various genes were detected after transfection in CNE1GL and CNE1 cells by qRT-PCR and western blot analysis. [file 1471-2407-13-124-S3.doc]

**Additional file 2**

**A**

**B**

**D**

**C**

Fig.1. The expressions of various genes were detected after transfection in CNE1GL and CNE1 cells by qRT-PCR and western blot analysis. (A) and (B) CNE1GL cells were transfected with siRNA-H3 or siRNA-mock for 72h. The mRNA and protein level of histone H3 were detected by qRT-PCR and western blot analysis. (C) CNE1 cells were cotransfected with LMP1 and mock, pcDNA6.0-H3 or pcDNA6.0-H3S10A for 36h. LMP1, histone H3 WT and mutant (H3S10A) expression were detected with antibodies against LMP1 and His epitope by western blotting. Detection of β-actin and histone H2A were used as loading controls. (D) CNE1GL cells were transfected with siRNA-MSK1 or siRNA-mock for 72h. The mRNA level of MSK1 was detected by qRT-PCR.
